# Supplementary material for: Training outcomes for audiology students using virtual reality or traditional training methods
Source: PLoS One. 2020 Dec 3;15(12):e0243380. doi: 10.1371/journal.pone.0243380 (PMC7714342; doi:10.1371/journal.pone.0243380)
Supplement: S1 Appendix — (DOCX) [file pone.0243380.s001.docx]

**Assessment of audiometric skills (20 questions)**

1. **You receive a patient for a hearing aid rehabilitation. You have consulted with the patient. What will be the first exam you will do? (single-choice question)**

❒ Weber test

❒ Speech audiometry

❒ Pure-tone audiometry

❒ In-vivo measurement

❒ Otoscopy

1. **Concerning the Weber test in a patient complaining of left-side hearing loss: (multiple-choice question)**

❒ It will be performed following pure-tone audiometry

❒ It will be performed following speech audiometry

❒ It will evoke a conductive hearing loss if it is lateralized on the left

❒ The tuning fork (or bone conduction vibrator) is placed on the mastoid

❒ It is part of acoumetric tests

**3- Concerning the general practices in audiometry: (multiple-choice question)**

❒ The blue color refers to the right ear

❒ To determine bone-threshold conduction thresholds, the bone conduction vibrator is placed on the mastoid process

❒ The hearing threshold selected is the lowest threshold for which the patient responds

❒ Pure-tone audiometry is expressed in dB SPL

❒ A medical history is not necessary

**4- Concerning pure-tone audiometry: (multiple-choice question)**

❒ The air conduction thresholds on the right are transcribed by the blue symbol “X.”

❒ It is better to determine high-pitched thresholds before low-pitched thresholds.

❒ Bone conduction thresholds are measured before air conduction thresholds

❒ It is preferable to begin measuring thresholds at 120 dB to get the patient used to it

❒ It is necessary to first determine the best ear thresholds

**5- Air or bone conduction thresholds determination will start at which frequency (single-choice question):**

❒ 250 Hz.

❒ 500 Hz.

❒ 1000 Hz.

❒ 2000 Hz

❒ 4000 Hz.

**6- Concerning speech audiometry: (multiple-choice question)**

❒ Speech audiometry is not essential.

❒ Speech audiometry is a reflection of social discomfort.

❒ Poor word recognition score (WRS) leads may indicate a retrocochlear disorder (e.g., vestibular schwannoma)

❒ The speech recognition threshold (SRT) corresponds to the softest level in dB HL at which the patient can repeat the word 50% in time

❒ The discrimination threshold corresponds to the percentage of words understood at 50 dB above the speech recognition threshold.

**7- If performing a Weber test on a patient who reports left-hearing loss: (multiple-choice question)**

❒ You do this at the end of the air- and bone-conduction threshold measurements

❒ An acoumetric Weber on the left side points to conductive hearing loss

❒ An acoumetric Weber on the left side points to a sensorineural hearing loss.

❒ The foot of the tuning fork must be placed on the right mastoid process

❒ The foot of the tuning fork must be placed on the left mastoid process

**8- Regarding this otoscopy, what is/are the true indicators(s)? (multiple-choice question)**

**
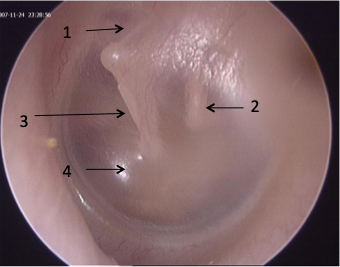
**

❒ 1 represents the pars flaccida

❒ 2 represents the malleus

❒ 3 represents the incus

❒ 4 represents the cone of light

❒ There is a retro-tympanic effusion

**9- Concerning masking for speech audiometry: (multiple-choice question)**

❒ It is never necessary.

❒ It is performed if there is a difference of more than 45 dB between the SRT and the pure-tone average in the contralateral ear.

❒ If masking is too low, there is a risk of overestimating threshold.

❒ Masking is performed using the bone conduction vibrator.

❒ Masking is performed before measuring bone conduction thresholds.

**10- Concerning aerial masking (multiple-choice question):**

❒ Air conduction masking for bone conduction thresholds is recommended if there is a 20 dB difference between the bone and air conduction thresholds.

❒ Air conduction masking for air conduction thresholds is recommended if there is a 50 dB difference between the air conduction threshold on one side and the contralateral bone conduction threshold.

❒ The masking value (Vm) is usually between 15 and 20 dB.

❒ The formula for calculating the minimum intensity (Imin) of bone conduction threshold masking is: I min = Intensity of sound tested - 50 dB + Rinne (i.e., difference in air and bone conduction threshold) of the masked ear + Vm.

❒ The formula for calculating the minimum masking intensity (Imin) of the air thresholds is: I min = Intensity of the sound tested - 50 dB + Rinne of the masked ear + Vm.

**11- Concerning masking in pure-tone audiometry (multiple-choice question):**

❒ It is between an effective intensity and a non-resounding intensity.

❒ It makes audiometry longer and more tiring for the patient.

❒ The formula for calculating the maximum intensity (Imax) of air conduction threshold masking is: I max = Intensity of the sound tested + 50 dB.

❒ The masking intensity rarely exceeds 90 dB.

❒ Too much masking is unpleasant but does not pose any risk to the patient.

**12- Concerning otoscopy (multiple-choice question):**

❒ The pars tensa forms most of the eardrum.

❒ The light triangle is located in front.

❒ The relief of the hammer handle is oblique at the top and forward.

❒ The pars flaccida is located at the posterior upper part of the eardrum.

❒ The round window is not visible for otoscopy.

**13- Concerning the following tympanograms: (multiple-choice question)**


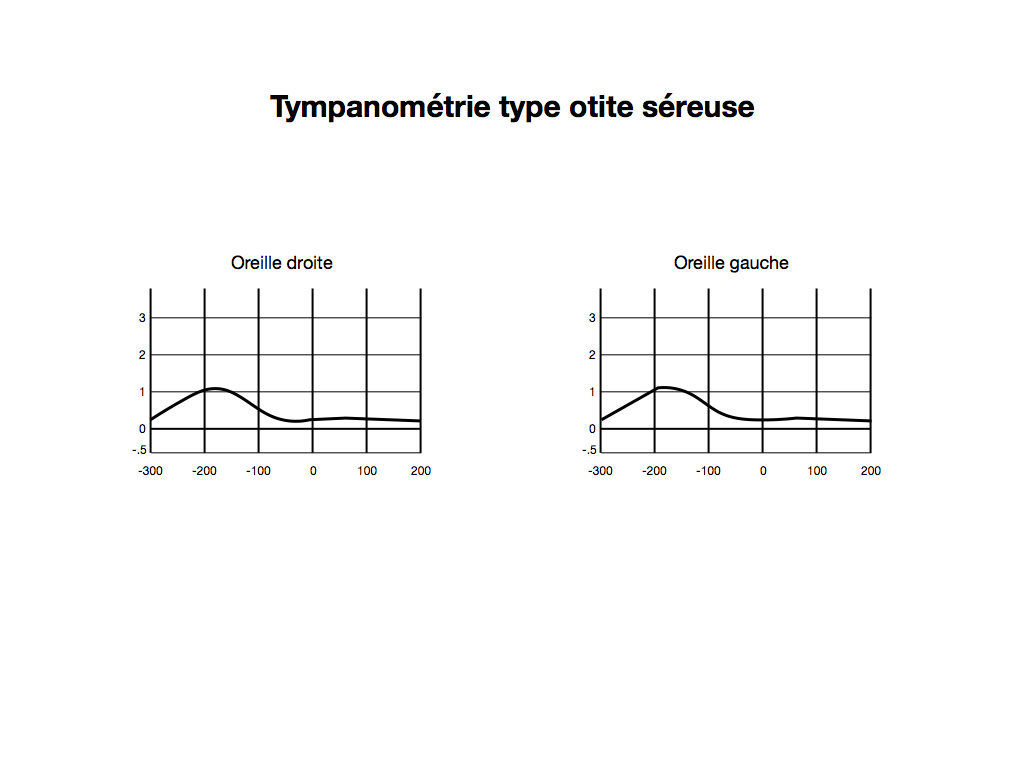


**1**


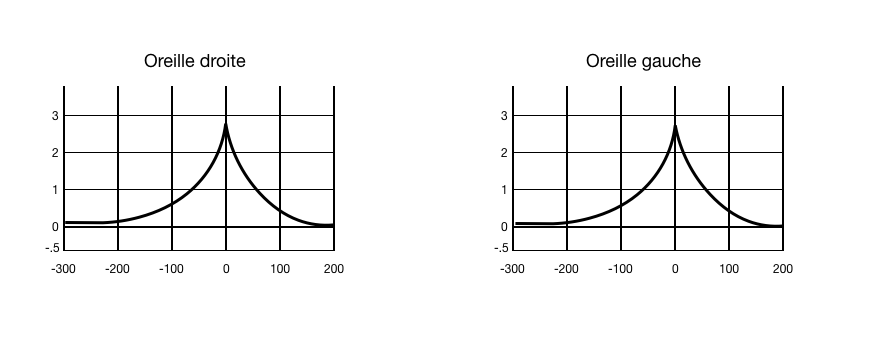


**2**


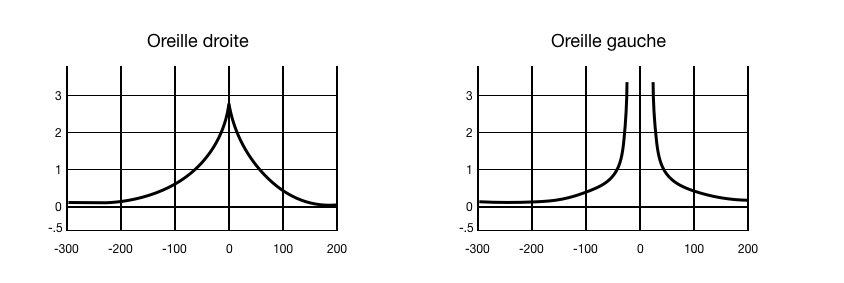


**3**

❒ N°1 refers to a left tubal dysfunction.

❒ N°2 refers to an increase in compliance of the right tympano-ossicular system.

❒ N°2 refers to a right tympanic perforation.

❒ N°3 refers to left otosclerosis.

❒ N°3 refers to a left ossicular dysfunction.

**
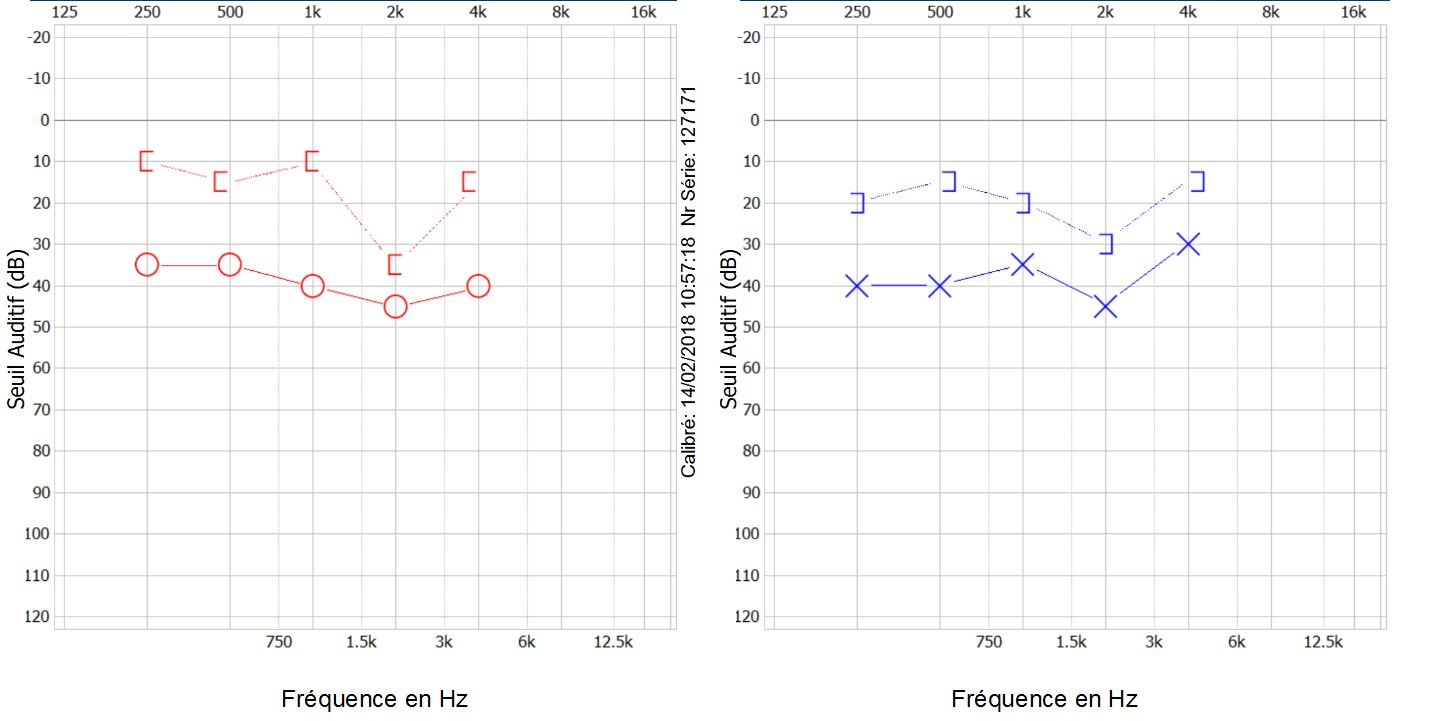
14- Concerning this pure-tone audiometry: (multiple-choice question)**

❒ This is a bilateral sensorineural hearing loss.
 ❒ This is a predominant conductive hearing loss on the left side.

❒ The bone threshold curve appears masked on each side.

❒ The audiometric Rinne at 1000 Hz is 25 dB on the left.

❒ This audiogram is compatible with bilateral otosclerosis.

**15- Concerning this pure-tone and speech audiogram for patient that is exposed to noise in**
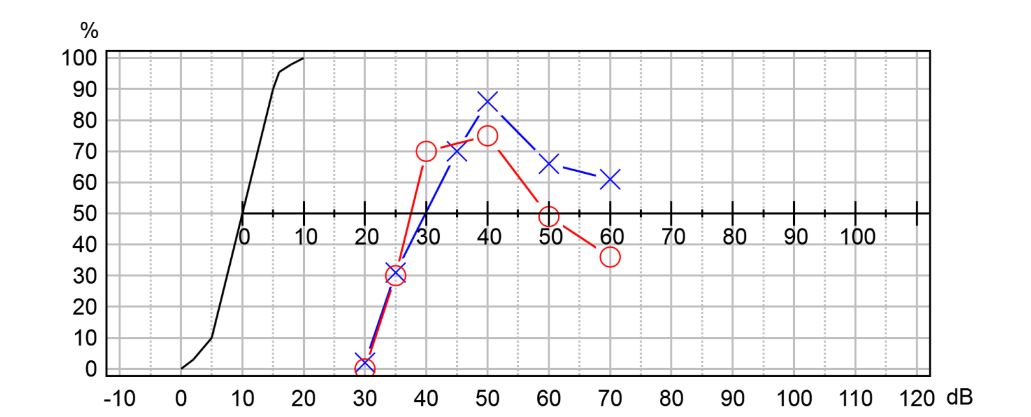

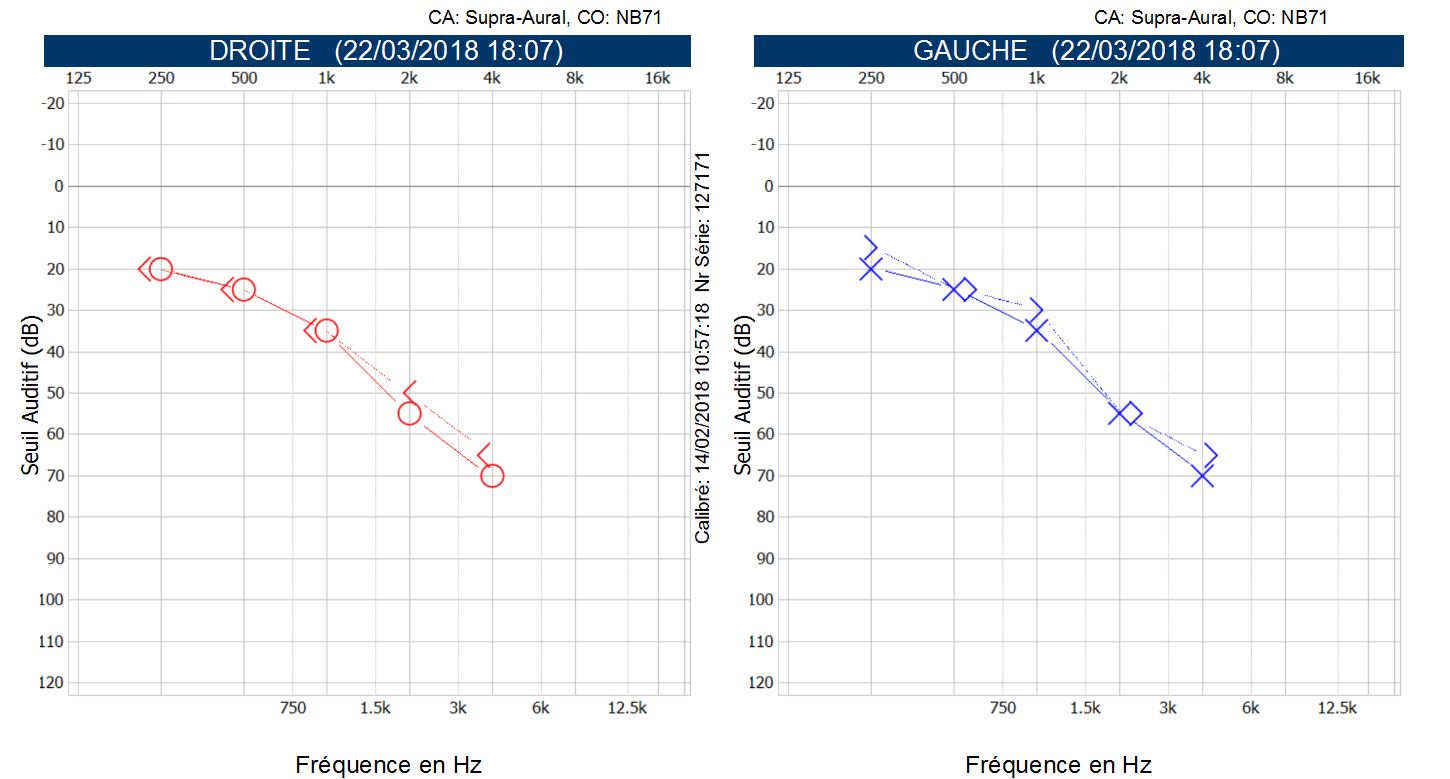
**their work environment (multiple-choice question):**

❒ The pure-tone audiometry curve is consistent with presbycusis.

❒ There is a distortion for the speech audiometry curve.

❒ The speech recognition threshold is 30 dB HL on the left.

❒ Masking should have been performed for speech audiometry.

❒ The discrimination threshold is 65 dB on the left.


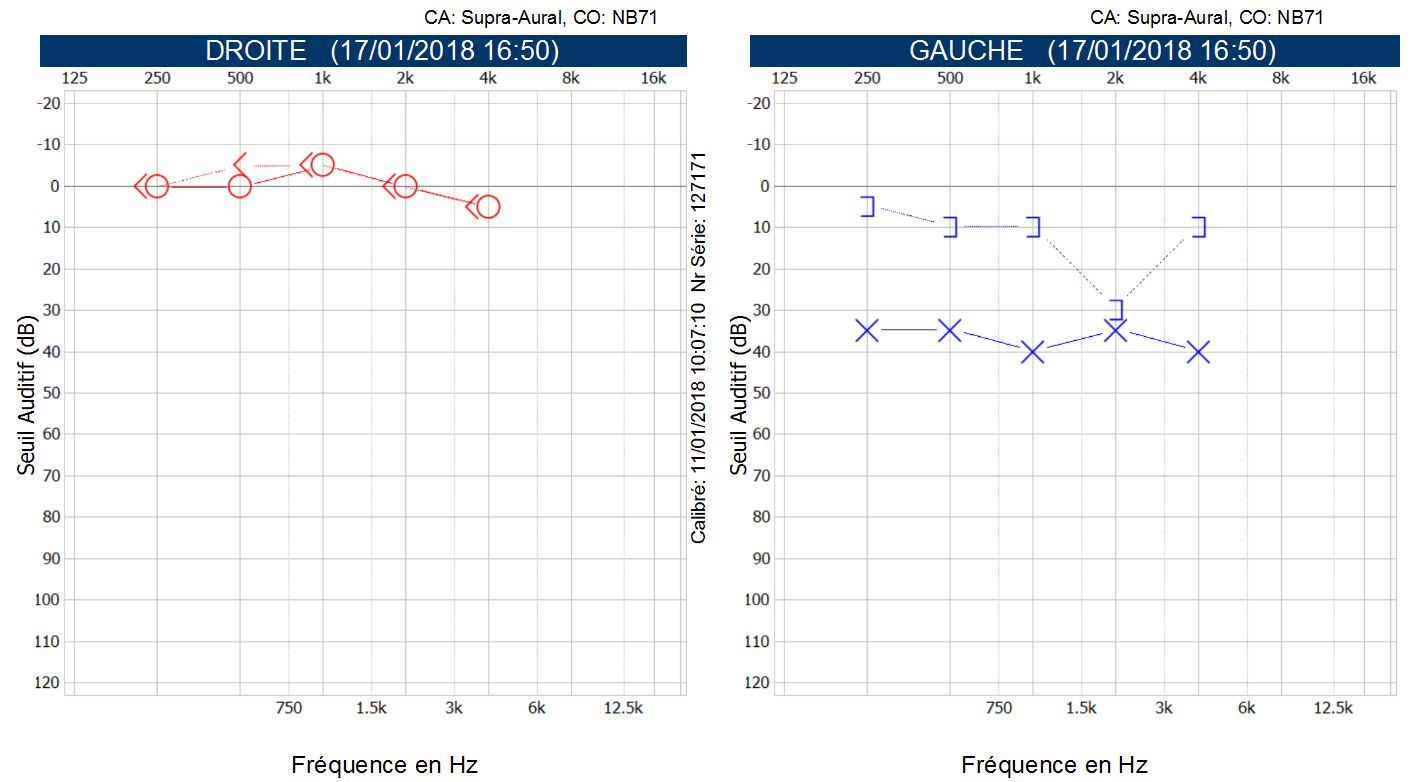
**16- For this audiogram, to measure a point on the left at 20 dB and 500 Hz in bone conduction, the masking value between (I min - I max) is: (single-choice question)**

❒ 30 dB

❒ 50 dB

❒ 60 dB

❒ 75 dB

❒ 80 dB

**17- Concerning this pure-tone and speech audiogram: (multiple-choice question)**

**
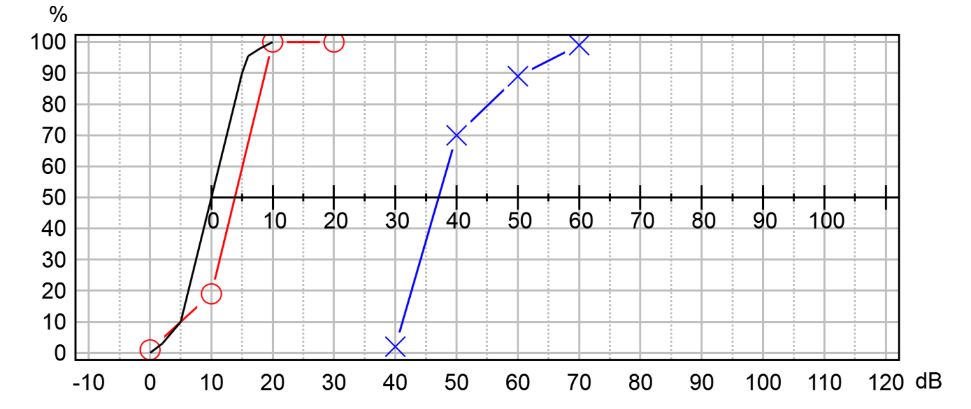

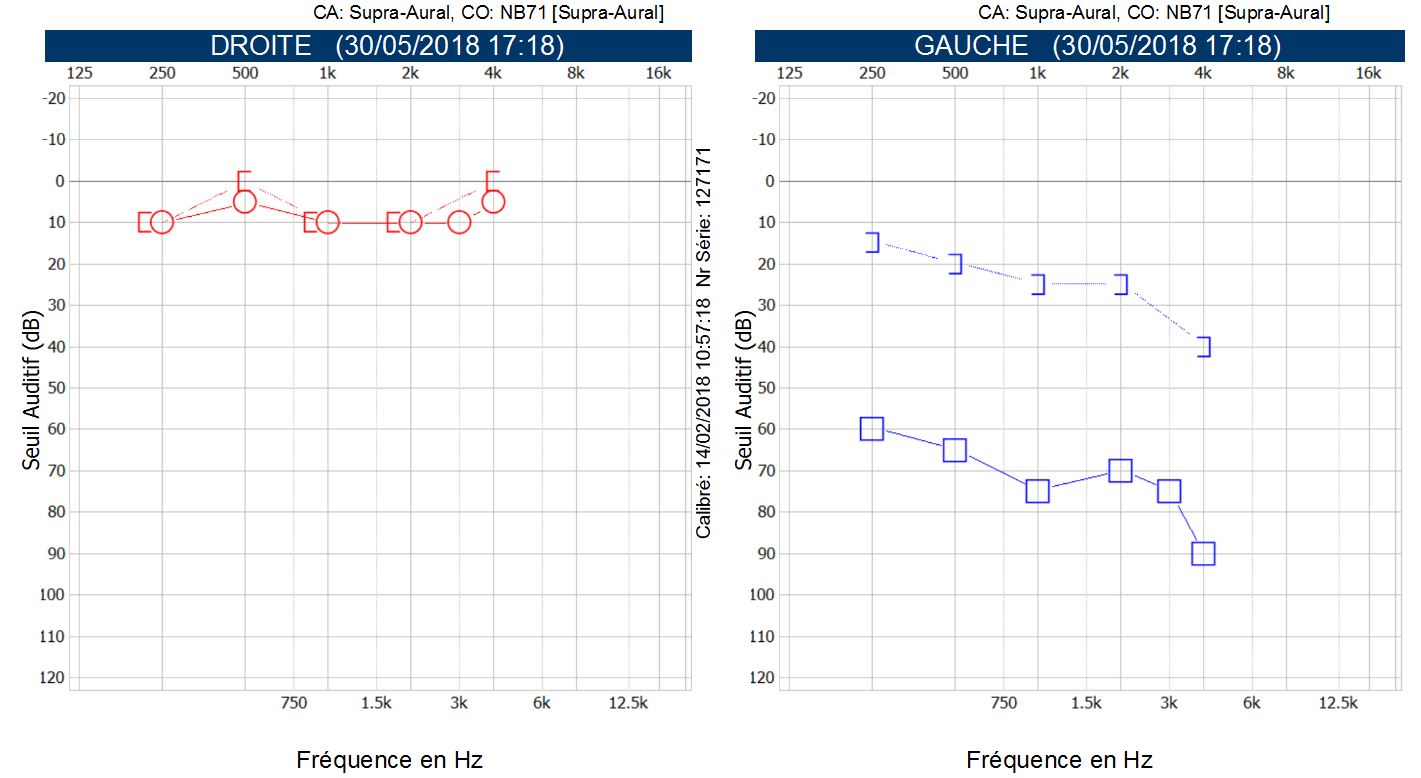
**

❒ There is a mixed hearing loss on the left.

❒ The speech recognition threshold on the left is 47 dB HL.

❒ The speech recognition threshold on the left is 37 dB HL.

❒ The speech audiometry curve is consistent with pure-tone audiometry.

❒ The speech recognition threshold on the right is 4 dB HL.

**18- Concerning this audiogram: (multiple-choice question)**


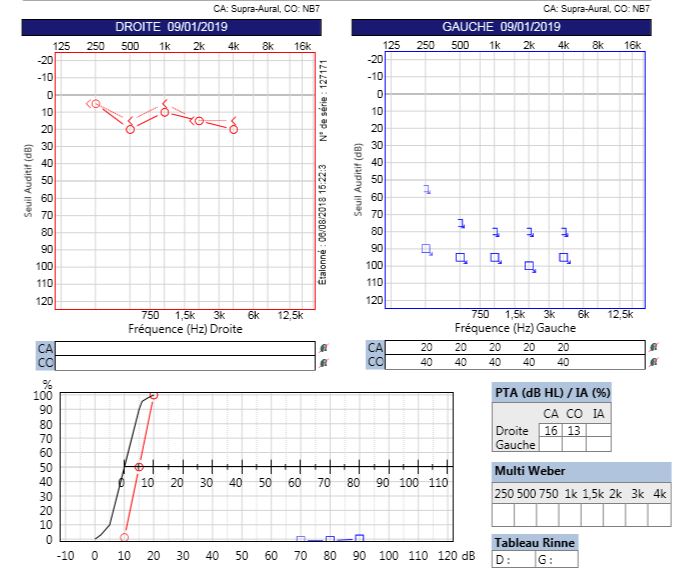

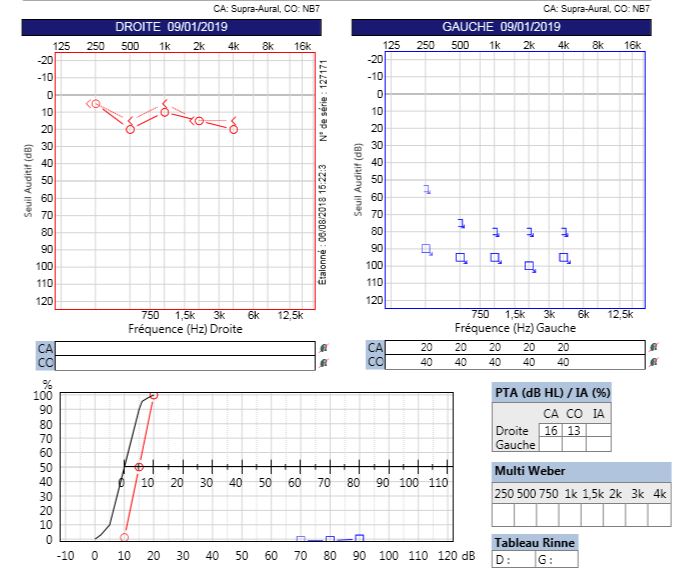


❒ There is right-side cophosis.

❒ The speech recognition threshold on the left is 0 dB HL.

❒ Masking of the right ear is not necessary to measure the left ear thresholds.

❒ The speech audiometry curve is consistent with pure-tone audiometry.

❒ The speech recognition threshold on the right side is 5 dB HL.

1**9- For this audiogram, to measure a right point at 10 dB and 4000 Hz in bone conduction,
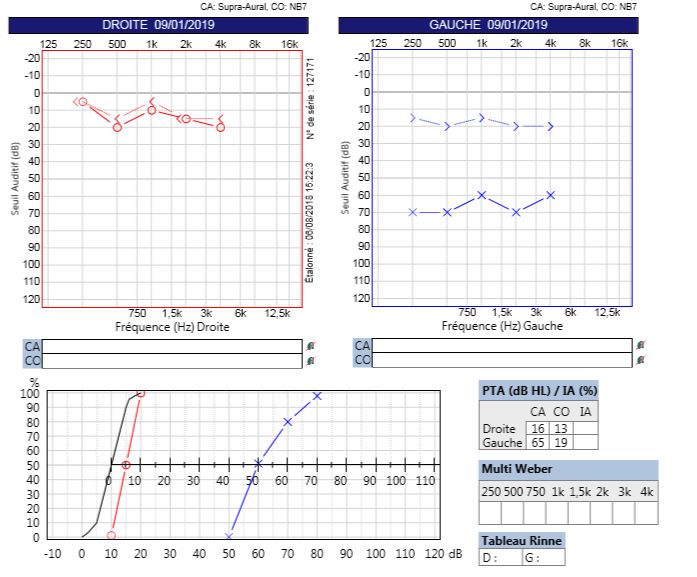
the minimum intensity for which masking is effective (I min) is: (single-choice question)**

❒ 45 dB.

❒ 65 dB.

❒ 75 dB.

❒ 85 dB.

❒ 95 dB.

**20- Concerning this audiogram: (multiple-choice question)**


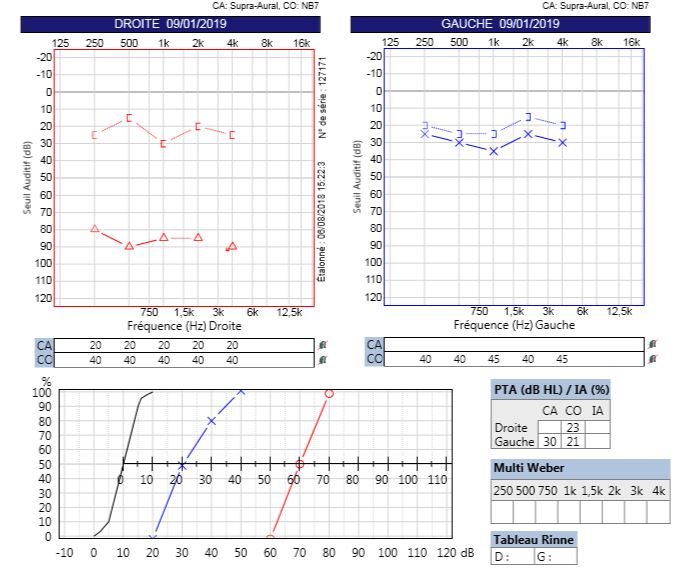

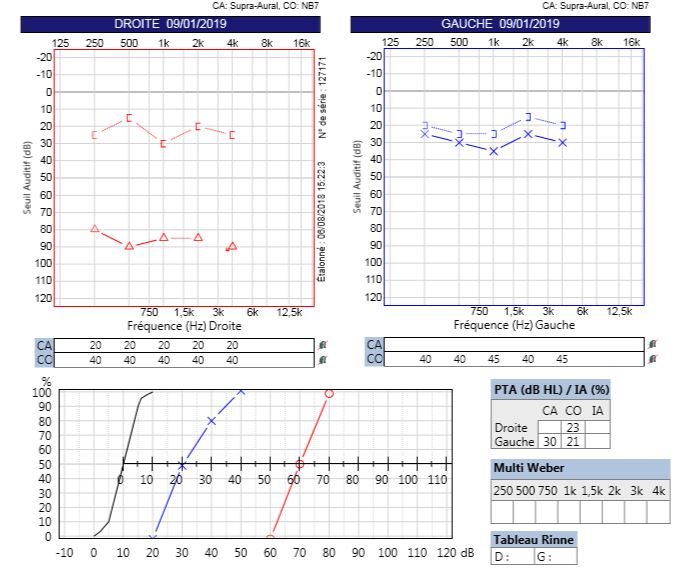


❒ There is a right-side cophosis.

❒ The speech recognition threshold on the left side is 60 dB HL.

❒ Masking of the right ear in speech audiometry is not necessary.

❒ The speech audiometry curve is consistent with pure-tone audiometry.

❒ The 4000 Hz right ear threshold in air conduction is not found.
