# Supplementary material for: Training outcomes for audiology students using virtual reality or traditional training methods
Source: PLoS One. 2020 Dec 3;15(12):e0243380. doi: 10.1371/journal.pone.0243380 (PMC7714342; doi:10.1371/journal.pone.0243380)
Supplement: S2 Appendix — (DOCX) [file pone.0243380.s002.docx]

**Évaluation des compétences en audiométrie (20 questions)**

**1- Vous recevez un patient pour une réhabilitation audioprothétique. Vous lui avez effectuer l’interrogatoire. Quel va être le premier examen que vous allez effectuer:**

❒ Le weber acoumétrique

❒ L’audiométrie vocale

❒ L’audiométrie tonale

❒ La mesure in vivo

❒ L’otoscopie

**2- Concernant le test de weber, chez une patiente se plaignant d’une hypoacousie gauche**

❒ il se fait après l’audiométrie tonale

❒ il se fait après l’audiométrie vocale

❒ il évoque une surdité de transmission si il est latéralisé à gauche

❒ le pied du diapason se met sur la mastoïde

❒ il fait partie des test d’acoumétrie

**3- Concernant les généralités en audiométrie, (cocher les propositions vraies) :**

❒ La couleur bleue désigne l’oreille droite.

❒ L’étude des seuils osseux se fait avec le vibrateur placé sur la mastoïde.

❒ Le seuil auditif retenu est le seuil le plus faible pour lequel le patient répond.

❒ L’audiométrie tonale est exprimée en dB SPL.

❒ La réalisation d’un interrogatoire n’est pas indispensable.

**4- Concernant l’audiométrie tonale (cocher les propositions vraies) :**

❒ Les seuils de conduction tonale aérienne à droite sont transcrits par le signe X.

❒ Il est préférable de rechercher les seuils aigus avant les seuils graves.

❒ La mesure de la conduction osseuse est réalisée avant celle de la conduction aérienne.

❒ Il est préférable de débuter la recherche des seuils à 120 dB pour habituer le patient.

❒ Il faut commencer par la recherche des seuils de la meilleure oreille.

**5- Concernant l’audiométrie tonale, il est préférable de commencer par la mesure de l’intensité (cocher la proposition vraie) :**

❒ 250 Hz.

❒ 500 Hz.

❒ 1000 Hz.

❒ 2000 Hz

❒ 4000 Hz.

**6- Concernant l’audiométrie vocale (cocher les proposition vraies) :**

❒ Sa réalisation n’est pas indispensable.

❒ L’audiométrie vocale est un reflet de la gêne sociale.

❒ Un trouble de l’intelligibilité fait suspecter une atteinte rétro-cochléaire ou des troubles auditifs centraux.

❒ Le seuil d’intelligibilité correspond à 50% des mots compris sur la courbe d’audiométrie vocale, et s’exprime en dB HL.

❒ Le seuil de discrimination correspond au pourcentage de mots compris 50 dB au dessus du seuil d’intelligibilité.

**7- Vous effectuez un test de Weber chez un patient se plaignant d’une hypoacousie gauche (cocher les proposition vraies) :**

❒ Vous l’effectuez à la fin de la mesure des seuils auditifs.

❒ Un Weber acoumétrique latéralisé à gauche oriente vers une surdité de transmission.

❒ Un Weber acoumétrique latéralisé à gauche oriente vers une surdité de perception.

❒ Le pied du diapason doit être posé au niveau de la mastoïde droite.

❒ Le pied du diapason doit être posé au niveau de la mastoïde gauche.

**8- Concernant cette otoscopie, quelle(s) est (sont) les proposition(s) vraie(s):
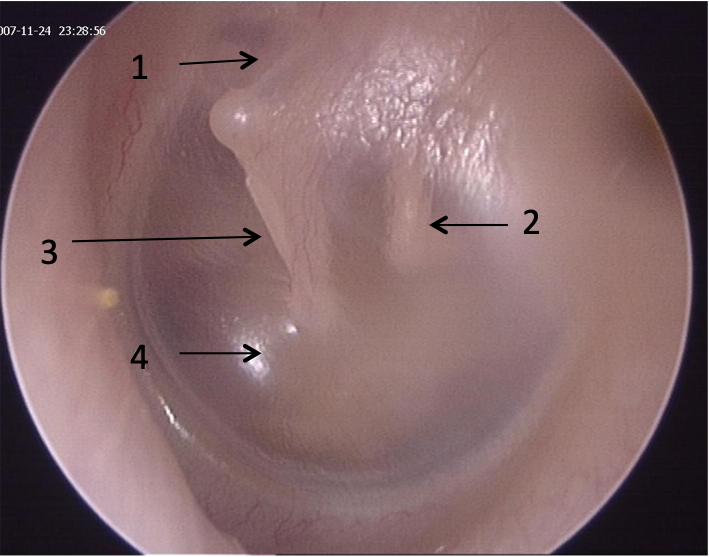
**

❒ Le 1 représente la pars flaccida

❒ Le 2 représente le malléus

❒ Le 3 représente l’incus

❒ Le 4 représente le triangle lumineux

❒ Il existe une épanchement retro-tympanique

**9- Concernant le masquage en vocale (cocher les propositions vraies) :**

❒ Il n’est jamais nécessaire.

❒ Il est effectué s’il existe une différence supérieure à 45 dB entre la vocale et la moyenne tonale en conduction osseuse de l’autre oreille.

❒ S’il est trop faible, il existe un risque de surestimer les seuils.

❒ Il se fait avec l’aide du vibrateur de conduction osseuse.

❒ Il se fait avant la recherche des seuils en conduction osseuse.

**10- Concernant le masquage aérien (cocher les propositions vraies) :**

❒ Un masquage aérien des seuils de conduction osseuse est recommandé en cas de différence de 20 dB entre les seuils osseux et aériens.

❒ Un masquage aérien de la courbe de conduction aérienne est recommandé s’il existe une différence de 50 dB entre le seuil aérien d’un côté et le seuil osseux controlatéral.

❒ La valeur du masque Vm est habituellement comprise entre 15 et 20 dB.

❒ La formule pour calculer l’intensité minimale (Imin) de masquage des seuils osseux est : I min = Intensité du son testé - 50 dB + Rinne de l’oreille masquée + Vm .

❒ La formule pour calculer l’intensité minimale (Imin) de masquage des seuils aériens est : I min = Intensité du son testé - 50 dB + Rinne de l’oreille masquée + Vm .

**11- Concernant le masquage en audiométrie tonale (cocher les proposition vraies) :**

❒ Il est compris entre une intensité efficace et une intensité non retentissante.

❒ Il rend l’audiométrie plus longue et fatigante pour le patient.

❒ La formule pour calculer l’intensité maximale (Imax) de masquage des seuils aériens est : I max = Intensité du son testé + 50 dB.

❒ L’intensité du masquage dépasse rarement 90 dB.

❒ Un masquage trop important est désagréable mais n’entraîne aucun risque pour le patient.

**12- Concernant l’otoscopie (cocher les propositions vraies) :**

❒ La pars tensa forme la majeure partie du tympan.

❒ Le triangle lumineux est situé en avant.

❒ Le relief du manche du marteau est oblique en haut et en avant.

❒ La pars flaccida est située à la partie postero-supérieure du tympan.

❒ La fenêtre ronde n’est pas visible à l’otoscopie.

**13- Concernant les tympanogrammes suivants (cocher les proposition vraies)**


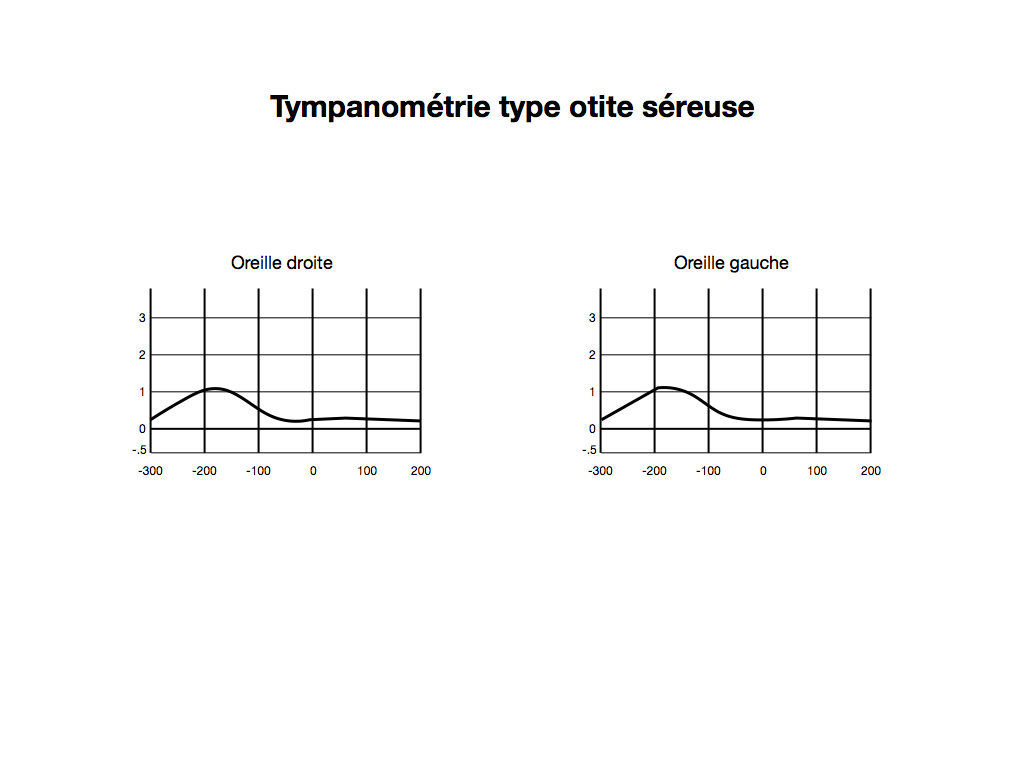


**1**


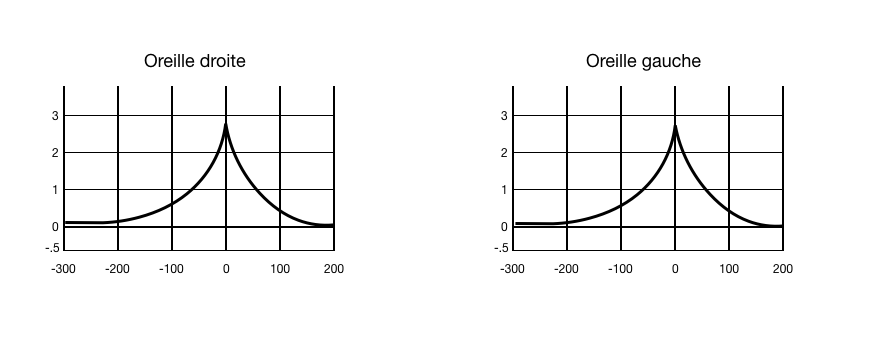


**2**


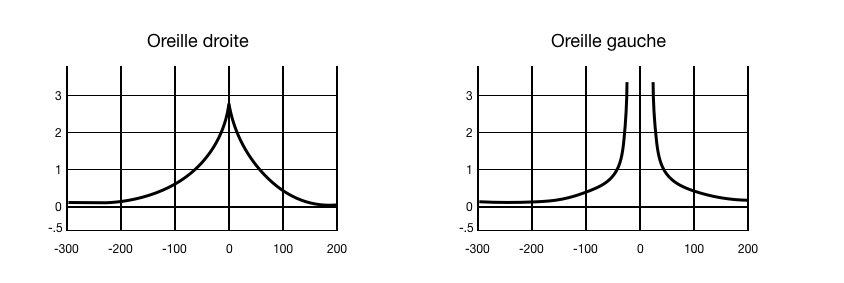


**3**

❒ Le N°1 évoque un dysfonctionnement tubaire gauche.

❒ Le N°2 évoque une augmentation de la compliance du système tympano-ossiculaire droit.

❒ Le N°2 évoque une perforation tympanique droite.

❒ Le N°3 évoque une otospongiose gauche.

❒ Le N°3 évoque une dysjonction ossiculaire gauche.

**14- Concernant cette audiométrie tonale (cocher les proposition vraies) :**

**
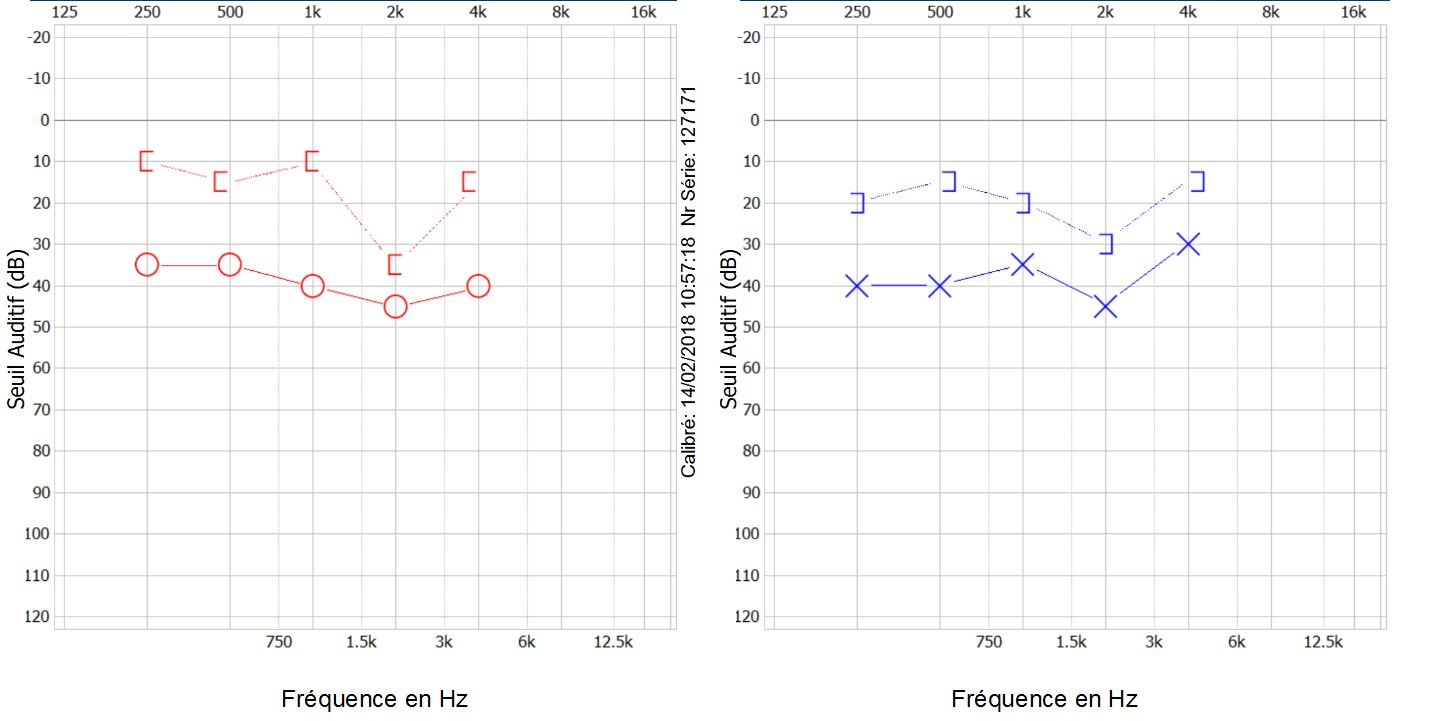
**

❒ Il s’agit d’une surdité de perception bilatérale.
 ❒ Il s’agit d’une surdité de transmission prédominant du côté gauche.

❒ La courbe osseuse apparaît masquée de chaque côté.

❒ Le Rinne audiométrique à 1000 Hz est de 25 dB à gauche.

❒ Cet audiogramme est compatible avec une otospongiose bilatérale.

**15- Concernant cette audiométrie tonale et vocale chez un patient exposé au bruit dans son milieu professionnel (cocher les proposition vraies) :**


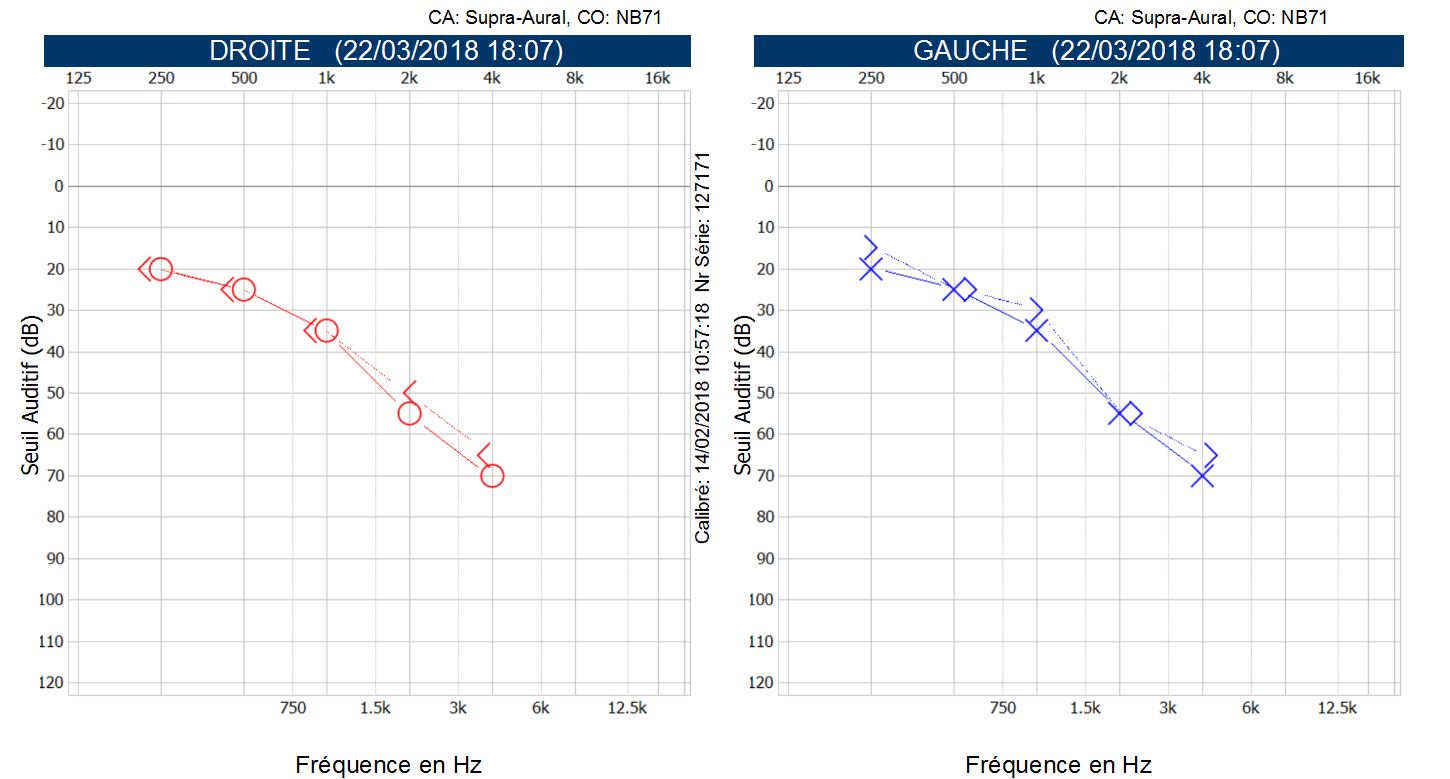

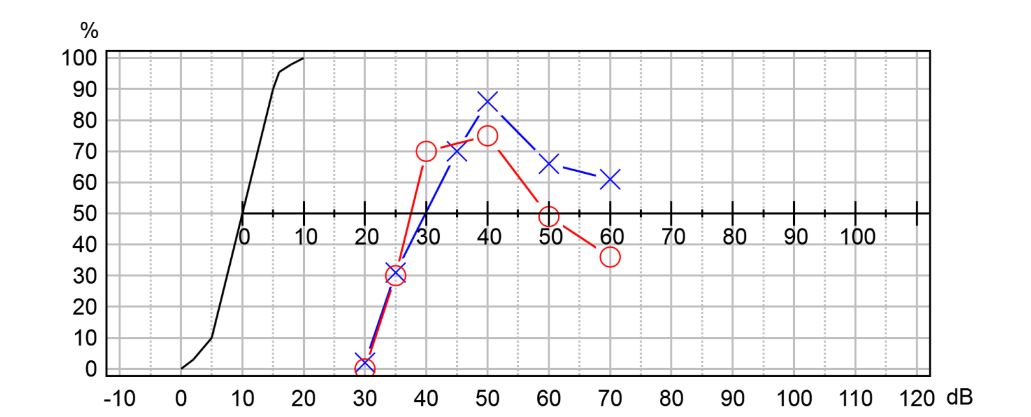


❒ La courbe d’audiométrie tonale est compatible avec une presbyacousie.

❒ Il existe une distorsion sur la courbe d’audiométrie vocale.

❒ Le seuil d’intelligibilité est de 30 dB HL à gauche.

❒ Un masquage aurait dû être réalisé pour l’audiométrie vocale.

❒ Le seuil de discrimination est de 65 dB à gauche.

**16- Concernant cette audiométrie, pour mesurer un point à gauche à 20 dB et 500 Hz en conduction osseuse, la valeur maximale de l’intensité (Imax) du masquage (delta**
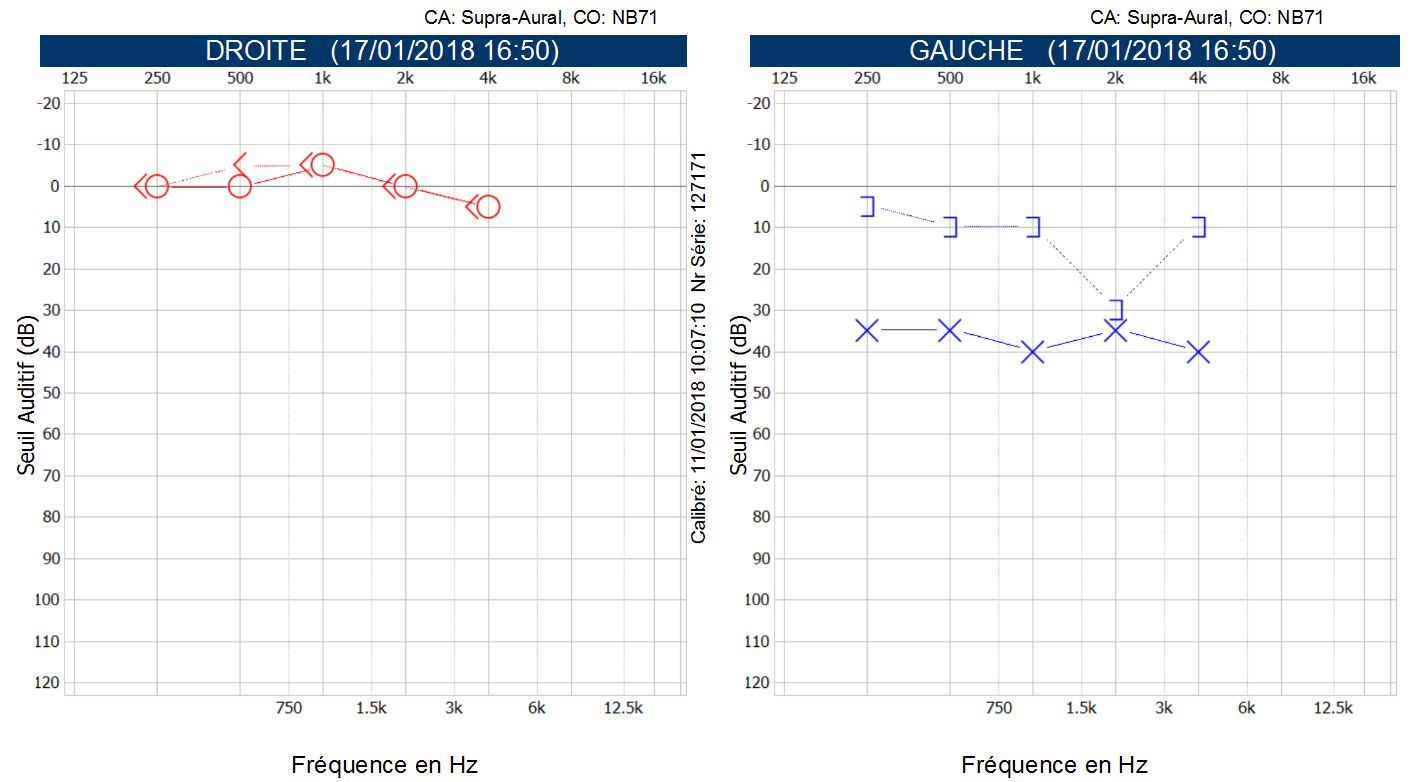
 **d’assourdissement à 15 dB, transfert transcrânien=60 dB) est : (cocher la proposition vraie)**

❒ 30 dB

❒ 50 dB

❒ 60 dB

❒ 75 dB

❒ 80 dB

**17- Concernant cette audiométrie tonale et vocale (cocher les proposition vraies) :**

**
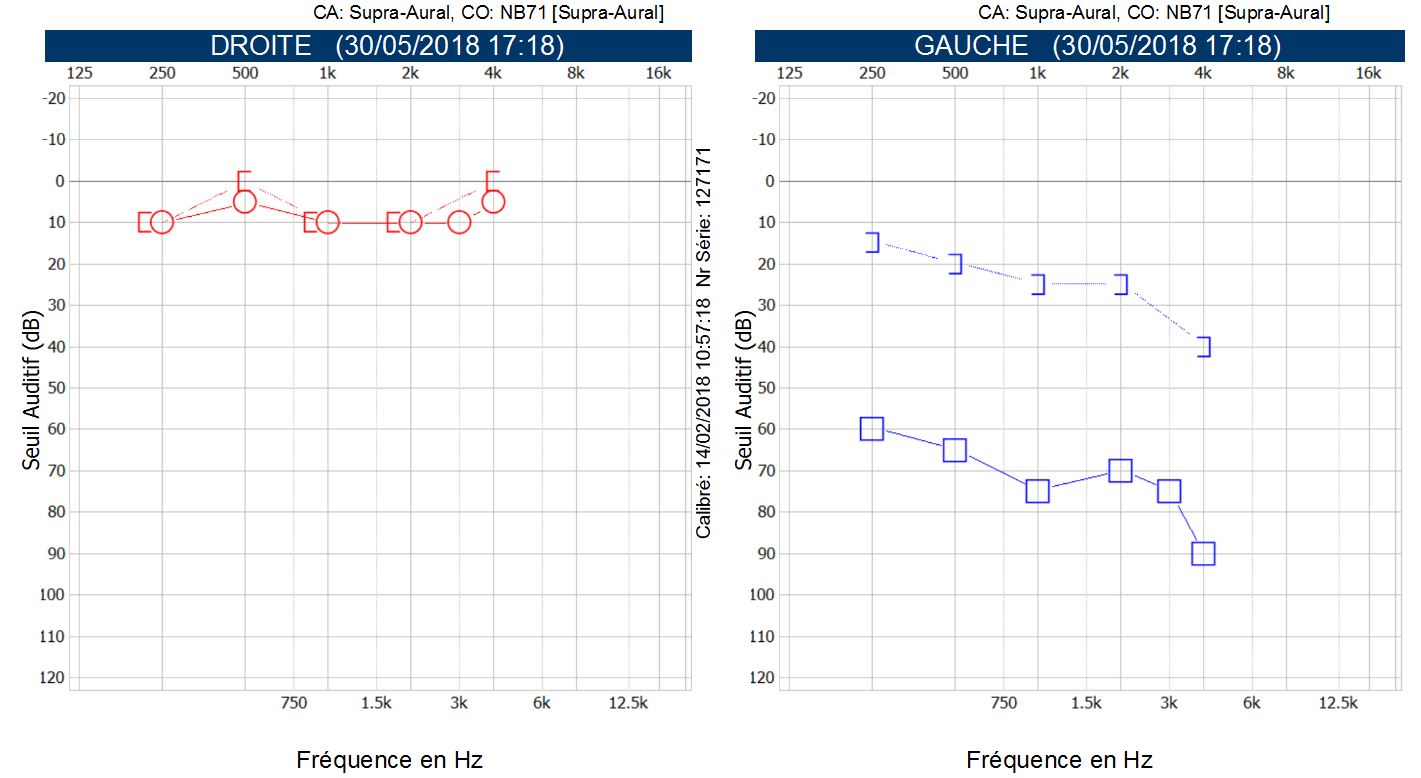

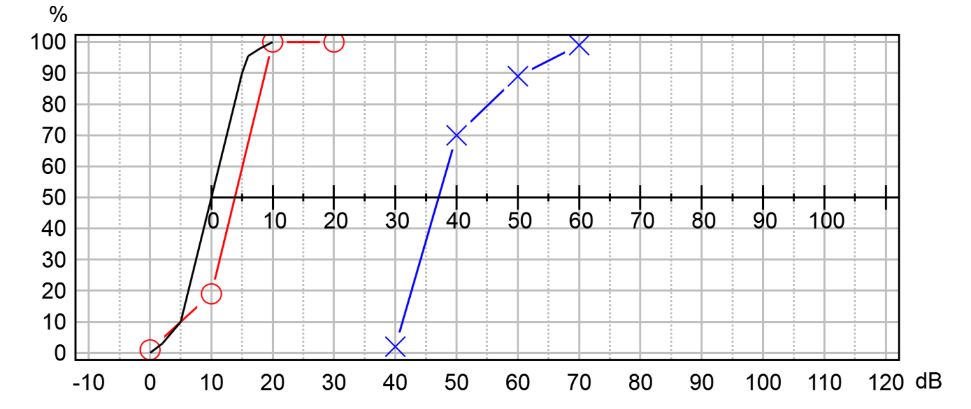
**

❒ Il existe une surdité mixte gauche.

❒ Le seuil d’intelligibilité à gauche est de 47 dB HL.

❒ Le seuil d’intelligibilité à gauche est de 37 dB HL.

❒ La courbe d’audiométrie vocale est concordante avec l’audiométrie tonale.

❒ Le seuil d’intelligibilité à droite est de 4 dB HL.

**18- Concernant cette audiométrie (cocher les propositions vraies):**


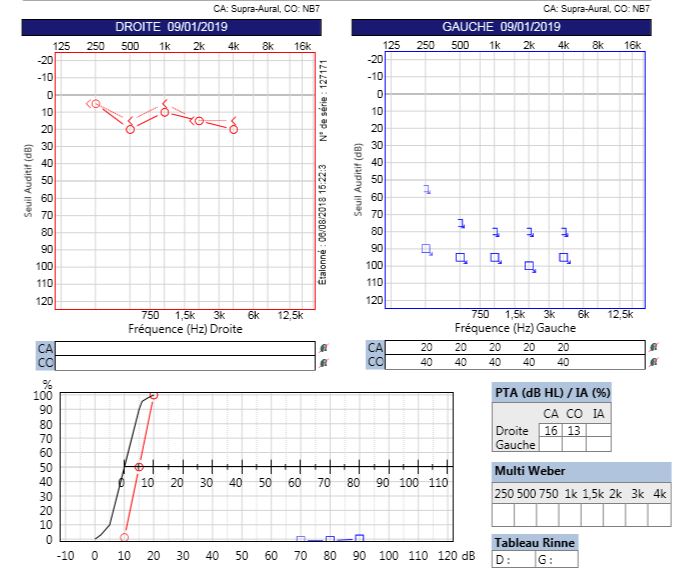

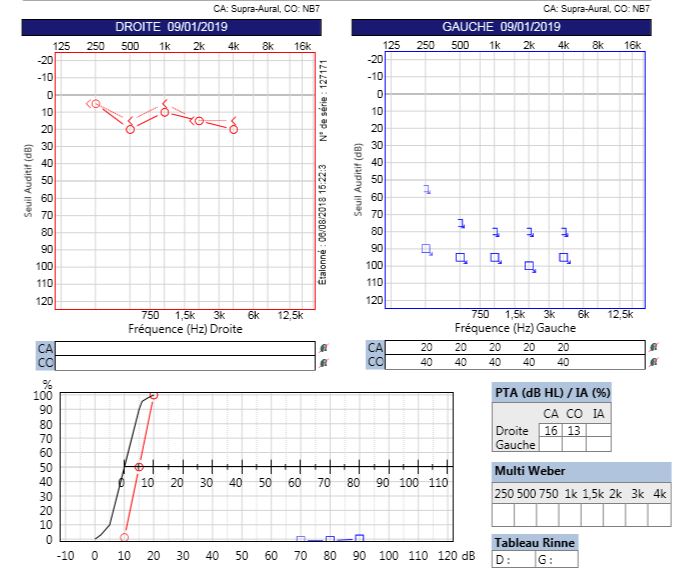


❒ Il existe une cophose droite.

❒ Le seuil d’intelligibilité à gauche est de 0 dB HL.

❒ Le masquage de l’oreille droite n’est pas nécessaire pour mesurer les seuils à gauche.

❒ La courbe d’audiométrie vocale est concordante avec l’audiométrie tonale.

❒ Le seuil d’intelligibilité à droite est de 5 dB HL.

**19- Concernant cette audiométrie, pour mesurer un point à droite à 10 dB et 4000 Hz en conduction osseuse, l’intensité minimale pour laquelle le masquage est efficace (I min) est (cocher la proposition vraie):**

**
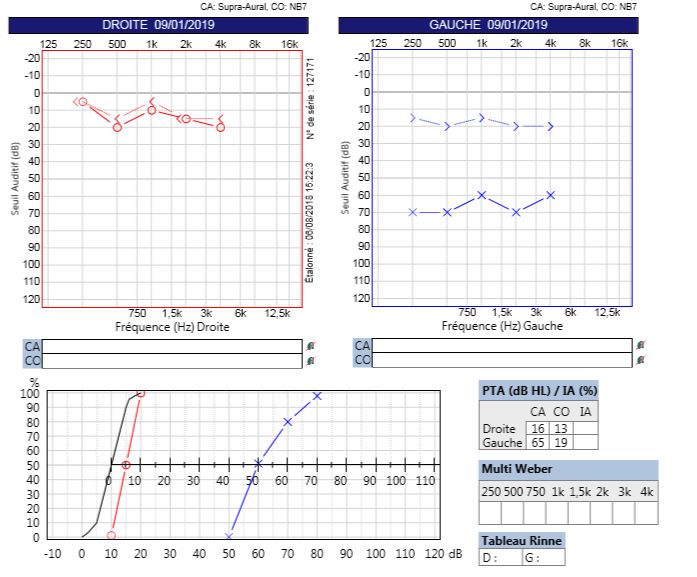
**

❒ 45 dB.

❒ 65 dB.

❒ 75 dB.

❒ 85 dB.

❒ 95 dB.

**20- Concernant cette audiométrie (cocher les proposition vraies):**


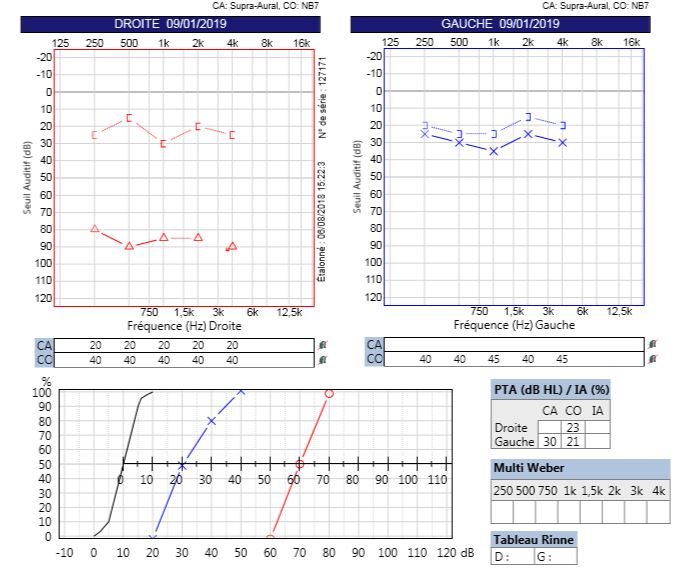

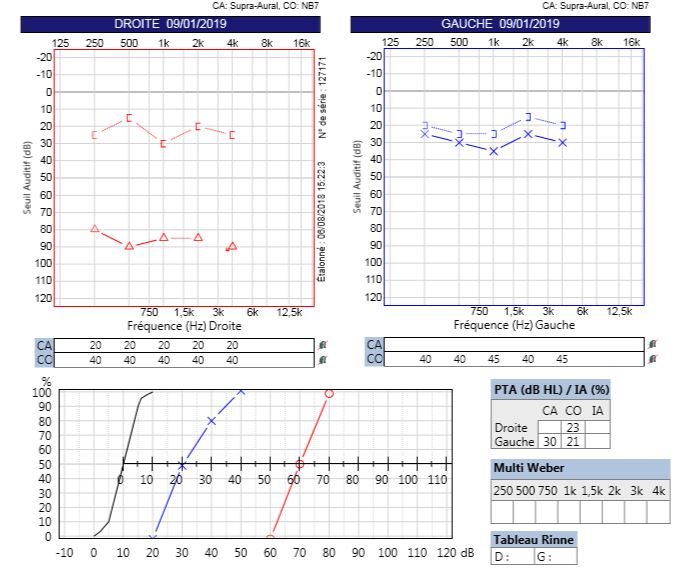


❒ Il existe une cophose droite.

❒ Le seuil d’intelligibilité à gauche est de 60 dB HL.

❒ Le masquage de l’oreille droite en audiométrie vocale n’est pas nécessaire.

❒ La courbe d’audiométrie vocale est concordante avec l’audiométrie tonale.

❒ Le seuil 4000 Hz droit en conduction aérienne n’est pas retrouvé.
