# Supplementary material for: Training outcomes for audiology students using virtual reality or traditional training methods
Source: PLoS One. 2020 Dec 3;15(12):e0243380. doi: 10.1371/journal.pone.0243380 (PMC7714342; doi:10.1371/journal.pone.0243380)
Supplement: Attachment — Submitted filename: VR-audiology-2020_FV_PLOS _ Reviewer Edits.docx [file pone.0243380.s004.docx]

**Virtual reality versus traditional audiometry training for audiology students**

Bakhos D^1, 2, 3^; Galvin J^4^; Aoustin JM^1, 6^ Robier M^1, 6^; Kerneis S^1^; Bechet G^5^; Montembault N^5^; Laurent S^5^; Godey B^5, 7^; Aussedat C^1, 2^

^1^CHU de Tours, service ORL et Chirurgie Cervico-Faciale, Boulevard Tonnellé, 37044 Tours, France.

^2^Faculté de Médecine de Tours, Université François-Rabelais de Tours, CHRU de Tours, 2, Boulevard Tonnellé, 37044 Tours, France.

^8^INSERM UMR 1253 I-brain, Université François-Rabelais de Tours, CHRU de Tours, 2, Boulevard Tonnellé, 37044 Tours, France.

^4^House Ear Institute, Los Angeles, California, USA.

^5^Ecole d’audioprothèse JE Bertin, Université de Rennes 1. Z.A de la grande marche. 35133 Javene, France

^5^Service d’ORL et Chirurgie Cervico-Faciale, CHU de Rennes, 2 boulevard Tonnellé, 37044 Tours, France

^6^Audilab, France

^7^Service d’ORL et Chirurgie Cervico-Faciale, CHU de Rennes, 2 boulevard Tonnellé, 37044 Tours, France

Corresponding Author:

David Bakhos

Service ORL et Chirurgie Cervico-Faciale

CHU de Tours

2 boulevard Tonnellé

37044 Tours

France

Phone number: +33 247 474 728

Fax: 0033 247 473 600

E-mail: [david.bakhos@univ-tours.fr](mailto:bakhos_d@med.univ-tours.fr)

**Abstract**

**Introduction**: Due to limited space and resources, it can be difficult to train students on audiological procedures adequately. In the present study, we compared audiology training outcomes between the traditional approach and a recently developed immersive virtual reality (VR) approach in first-year audiology students.

**Participants and method**: Twenty-nine first-year audiology students participated in the study; 14 received traditional training, and 15 received the VR training. Pre- and post-training evaluation included a 20-item test developed by an audiology educator. Post-training satisfaction and self-confidence were evaluated using Likert scales.

**Results**: Mean post-training test scores improved by 6.9±9.8 percentage points with traditional training and by 21.1±7.8 points with the VR training. After completing the traditional training, students were trained with the VR system, in which the mean scores improved by an additional 7.5 points. The mean satisfaction rating was 74% for the traditional training and 100% for the VR training, and the mean self-confidence rating was 71% for the traditional training and 92% for the VR training.

**Conclusion**: These results suggest that a VR training approach may be an effective alternative or supplement to traditional training for audiology students

**Keywords**: Audiology; education; training; virtual reality

**Introduction**

Training in audiometric procedures and diagnosis is an essential part of educating audiology students. Audiometric procedures include mathematical, physiological, and psychophysical rules. For example, when measuring audiometric thresholds, it is important to know the appropriate levels of contralateral noise masking to avoid misdiagnosis or, more importantly, overstimulation. In France, during the first year of audiology education, audiometry training involves theoretical course work (80 hours), lectures, and clinical training. Traditional clinical training is performed using tutorials (3 hours), where students will train with each other or sometimes train with volunteers that have normal hearing or hearing loss. During the second and third years, the students will practice under the direct and indirect supervision of a licensed practitioner during their internship. Given the number of students, as well as limited resources in terms of the training time and space available, this supervision is not always possible, however. . In addition, such training, even when supervised, does not meet the recommendation by the French National Authority for Health that students be sufficiently trained before they are allowed to work with patients or volunteers (1).

Simulations have become an increasing part of medical education. Using simulators has been shown to improve patient safety and reduce costs and morbidity (2). Various computer-assisted simulators have been developed in recent years to support health professional training. These models have been created to improve the experience for the learner before medical students interact with human patients or volunteers. In this context, simulators are strongly supported by supervisors in terms of students’ acquisition of both technical and non-technical skills (3). Using simulators to train audiometry will better prepare students for future clinical practice. Over the last two decades, simulations have been increasingly integrated into medical education to facilitate the acquisition of knowledge and practice in a safe environment, allowing students to train and learn from their mistakes without risk to patients (8). Simulators allow students to repeatedly learn and practice until they feel confident interacting with a patient. Currently, there are few simulators directed at developing students’ audiometry skills.

One area of recent development is the use of virtual reality (VR) simulators, which allow for a more immersive experience for the learner. Given great advances in computer technology, VR simulators for the medical field have greatly improved. VR simulators have been shown to improve learning outcomes for various surgery training steps. They have been used to train clinical reasoning in the field of traumatology to enhance decision-making among students (4-7). We recently developed a VR simulator for audiometric training (9) that simulated the clinical environment. Seven clinical cases were simulated, including otosclerosis, presbycusis, vestibular schwannoma, incus luxation, malingering presentation, sudden idiopathic deafness, and bilateral hearing loss due to cholesteatoma. For each clinical case, “beginner” and “expert” modes were created. The beginner mode provides feedback during the training session to inform students of errors and to provide correction; expert mode provides no feedback. At the end of the VR training session, a report is produced that summarizes the errors made for each clinical case. External experts (medical educators and licensed practitioners) and otolaryngology students reported satisfactory validity.

The initial evaluation of the VR simulator involved expert educators and otolaryngology students that had already experienced traditional audiometry training. However, it is still unclear how training outcomes may differ between a traditional or a VR simulation approach. In this study, we compared training outcomes between a group of audiology students that received traditional audiometry training or a single session of VR training. We also evaluated students’ satisfaction and self-confidence with the two training approaches.

**Participants and methods**

We conducted a prospective single-center study with parallel groups at the Fougeres School of Audiology in France. Thirty-one first-year students were recruited for the study; two were excluded because they had repeated their first year. Accordingly, 29 students participated in the study (mean age: 21.1±4.1 years). The study was conducted in December 2019, after students had completed all theoretical course work for audiometry. None of the participants had yet begun an internship at a hospital or audiology center. All data were anonymized prior to analysis. The ethics committee of Tours Hospital approved this project (2018-091).

Fourteen students (mean age: 20.4 ± 1.7 years) were included in the Traditional Training (TT) group. The TT group received 3 hours of training supervised by a teacher in the Audiology School. During the training, the teacher first reviewed basic audiometry principles, after which students were allowed to practice on the teacher who simulated presbycusis and unilateral deafness, and then practice on each other

Fifteen students (mean age: 21.7±5.6 years) were included in the Virtual Reality Training (VRT) group. The VR training was performed using the previously developed simulator (9). During the training, a supervisor first explained how the VR system works introduced the VR hardware, which included the headset, 2 captors, and handles (Oculus Rift ®). Next, each student was trained on 3 clinical cases (presbycusis, vestibular schwannoma, and sudden idiopathic deafness) using the beginner mode, which provided feedback during the session. Next, audiometry diagnosis and management were evaluated for each of the clinical cases, and a report was generated that summarized the errors during the evaluation. The duration of training for each case was approximately 30 minutes, and the total time of the VR training session was approximately 3 hours, including the 20 minutes of introducing the system and 20-minute breaks between clinical cases to avoid fatigue. Figure 1 shows screenshots of the VR system.

---------------------------------------Insert figure 1-----------------------------------------------

The evaluation was developed by experts in audiology teaching (i.e., authors MN, LS in the present study), and included 20 questions regarding otoscopy, tone and speech audiometry, acoumetry, masking and tympanometry based on the audiometry theoretical course work and lectures; an English translation of the evaluation can be found in Appendix 1. The scoring for each question was weighted depending on the number of errors: 1 point in case of no error, 0.5 points in case of 1 error, 0.3 in case of 2 errors, and no points in the case or 3 or more errors. The maximum possible score was 20, and scores were converted to percent correct. After completing the posttest, students in the TT group performed the same VR training as for the VRT group and were then re-tested.

After completing the TT or VR training, post-training satisfaction and self-confidence were evaluated using subscales with 5-point Likert response (1 = “Strongly disagree”, 2 = “Disagree”, 3 = “Indifferent”, 4 = “Agree”, 5 = “Strongly agree”). For satisfaction, the five items included the realism of training, feedback, support, comprehension, and degree of complement to the theoretical lessons. For self-confidence, the six items included consultation, otoscopy interpretation, audiometry thresholds determination, speech audiometry procedures, masking, and overall confidence for clinical interaction with a patient. After training with the VR simulator, a four-item subscale with a 5-point Likert response was also administered to evaluate the immersive and realistic aspects of the VR simulator in relation to theoretical lessons and online software. Overall satisfaction or self-confidence ratings were calculated, and scores ≥ 4 were considered satisfactory. Students completed the evaluation at the beginning of the training session; at the end of the session, they completed the evaluation and the surveys.

**Results**

Figure 2 shows student scores before and after the training; mean scores are indicated by the horizontal lines. The mean pre-test score was 41.2±10.2 percent correct for the TT group and 39.5±6.5 percent accurate for the VRT group. No significant differences in pre-test scores were observed between groups (U=87; p=0.444). The mean posttest score for the TT group significantly improved to 48.1±8.7 percent correct (W=72; p=0.021). Following the subsequent VR training, the mean TT posttest score further improved to 55.6±10.9 percent correct, significantly better than pre-test scores (W=89; p=0.0029) and substantially better than posttest scores after the traditional training (W=89; p=0.00029). The mean posttest score for the VRT group significantly improved to 60.5±7.1 percent correct (W=120; p<0.001). The mean improvement relative to pre-test scores was significantly larger for VRT group (21.1±7.8 percentage points) than for the TT group (6.9 ±9.8 percentage points) (U=22.5; p<0.0001). After the subsequent VR training, there was no significant difference in post-test scores between the TT group and the VRT group (U= 66; p=0.0908).

---------------------------------------Insert figure 2-----------------------------------------------

Post-training satisfaction ratings were ≥ 4 in 74% of the TT group and 100% of the VRT group. Post-training self-confidence was ≥ 4 in 71% of the TT group and 92% of the VRT group. Figure 3 shows the results for each item of the surveys. Significant differences across training groups were observed for the realism of the audiological cases (U=45.5; p=0.0026), support during the training session (U=0; p<0.0001), the degree of complement to the theoretical courses (U=52.5; p=0.0022), confidence for speech audiometry (U=38.5; p=0.0017), and confidence regarding the use of masking (U=45; p=0.0048).

---------------------------------------Insert figure 3-----------------------------------------------

Figure 4 shows the ratings for the VR system after the VRT and TT groups completed the VR training. Compared to theoretical courses, students agreed or strongly agreed (i.e., scores ≥ 4) that the VR training was more realistic (93%) and immersive (100%) than theoretical course work. They also agreed or strongly agreed that the VR training was more realistic (53%) and immersive (67%) than online software. All students (100%) agreed or strongly agreed that the VR training was complementary to theoretical courses and would recommend VR training to their colleagues.

---------------------------------------Insert figure 4-----------------------------------------------

**Discussion**

The present study compared traditional audiometry training versus VR training for first-year audiology students. While post-test scores improved after training for both groups, the improvement for the VRT group was more substantial than for the TT group. These findings suggest that the tested VR simulator may be a useful tool for audiology education. VR training offers the possibility for the students to practice their skills in an extensive and repetitive manner with feedback but without any consequences to real patients or volunteers (10). We also found great enthusiasm for the VR simulator among the students tested.

Some previous studies have investigated interest in VR training versus traditional training in other medical fields. In general, VR training offered an advantage for learning theoretical knowledge over traditional, lecture-based education teaching of dentistry (11) and anatomy (12). In surgery, the goal of training is quite different because evaluation is based on the acquisition of technical skills. Some meta-analyses were conducted for gastro-intestinal endoscopy training (4, 5) and for surgery of the ear, nose, and throat (6) using VR. They showed that VR training alone allowed the similar acquisition of technical skills as with traditional training, and thus considered VR simulators to be a useful learning tool.

Feedback during training is essential to help students to improve performance, allowing students to learn from their mistakes quickly. In the present VR system, concurrent feedback in beginner mode is cued by a sound alarm in case of error, followed by explanations of why the response was wrong. In this study, VR training was performed only using beginner mode. In our VR system (but not used in the present study), expert mode provides no concurrent feedback, but rather terminal feedback at the end of the evaluation. Some studies have suggested that terminal feedback is more effective than concurrent feedback, given the guidance hypothesis (13), which indicates that while concurrent feedback may help to learn a skill, learners are not dependent on concurrent feedback (14). In the present study, students appreciated the concurrent feedback, with an overall satisfaction of 80% in the TT group after training with the VR simulator, and 100% in the VRT group. Only negative feedback was used in the VR training to indicate cases of error; positive feedback in cases of success has also demonstrated training benefits (15).

Traditional training for objective audiometry using normal-hearing volunteers, hard of hearing volunteers, or manikin simulators has demonstrated improved self-confidence among audiology and speech language pathology students (16, 17). This improved self-confidence has been also demonstrated after “boot-camps” (18), where students can practice with immediate feedback. Self-confidence as well as self-assessments play a major role in developing and improving clinical skills (19). In the present study, the students similarly exhibited greater self-confidence after the training sessions, especially in the VRT group.

There are different types of simulators, depending on their fidelity and resemblance to reality, divided into three main classes ranging from low- to high-fidelity (20). Non-computerized manikins correspond to low-fidelity simulators (<https://www.aheadsimulations.com/carl-for-training>). They are used to train hearing aid manipulation, the removal of cerumen, real-ear unaided response measurements, and high-gain hearing aid fitting (21, 22). Some computerized simulations allow training for specific tasks such as otoscopy, pure-tone air and bone conduction audiometry with online virtual patients (23). Some of these online solutions are freely available ([https://personalpages.manchester.ac.uk/](https://personalpages.manchester.ac.uk/staff/tim.wilding/PTA_Sim/index.html)), while others require payments. One high-fidelity simulator exists in the field of audiology (Intelligent Hearing Systems, Miami, FL), and is a computerized manikin to train objective audiometry (e.g., auditory brainstem response, otoacoustic emissions).

Some limitations to the present study should be discussed. First, our study was from a single center; however, a multi-center study to confirm results are needed. Second, we did not evaluate long-term retention of the training because students went on to internships at different audiology centers; it is possible that additional learning may have occurred at these centers making it difficult to ascertain training retention. Previous studies have shown no difference in terms of retention with traditional or VR training (24, 25). However, greater satisfaction and engagement was reported by students who received VR training (25). Similarly, greater satisfaction with VR training was reported in the present study, and students were very satisfied with the immersive and realistic environment. We note that some procedural learning may have been possible, given that the same test was given before and after training. Even if some procedural learning may have occurred, the post-training improvement was more than double for VRT group, compared to the TT group. After traditional training, the mean improvement for the TT group was 6.9 percentage points; after completing the subsequent VR training, the mean score improved by an additional 7.5 percentage points. However, even after the VR training (and possible procedural learning), the mean score for the TT group remained 4.9 percentage points below that of the VRT group (although the difference at these endpoints was not significant). For future implementation, it would be advisable to create different questionnaires that cover different aspects of the clinical cases across pre- and post-training testing.

The current VR simulator satisfies the directive from the French National Authority for Health and requires sufficiently trained students before work with patients or volunteers. Future development of VR simulators would greatly benefit the training of a broader range of clinical cases and procedures. For example, behavioral measures of auditory thresholds in children are challenging; future VR systems could train students on age-appropriate audiometric techniques (e.g., evaluating infant responses). Further studies could also be directed at norming VR evaluation scores across different levels of medical education. For example, it would be useful to know the range of scores for first-year and third-year students. Once this range is identified, the VR evaluation could also determine whether students have sufficiently progressed in their audiological education. VR simulators could also be used for continuing education and certification programs for practitioners. Further studies are needed to demonstrate the real-life benefit of VR training for practitioners and patients and to determine whether VR training can reduce the number of medical errors in clinical practice.

**Conclusions**

Simulations are widely used to train students in various fields of medicine; VR permits a safe training environment without risk to patients or volunteers. In this study, our immersive VR training system provided audiology students with better learning and self-confidence than found with traditional training. Currently, the VR simulator can be used as a supplement to traditional audiology training; additional studies are needed to know whether it can replace traditional training. Further technological developments are also needed to expand the audiology-training modules, such as behavioral and objective hearing tests for pediatric patients.

**Declarations**

Funding: Audilab funded the virtual reality system

Conflicts of interest: None

**References**

1. Granry JC, Moll MC. 2012. Rapport de mission. État de l'art (national et international) en matière de pratiques de simulation dans le domaine de la santé: Dans le cadre du développement professionnel continu et de la prévention des risques associés aux soins. [Mission report. State of the art (national and international) in health simulation practices: As part of ongoing professional development and prevention of risks associated with care. French National Authority for Health.]

2. Graafland M, Schraagen JM, Schijven MP (2012) Systematic review of serious games for medical education and surgical skills training. Br J Surg 99:1322–1330.

3. Yiasemidou M, Glassman D, Tomlinson J, Song D, Gough MJ (2017) Perceptions about the present and future of surgical simulation: A national study of mixed qualitative and quantitative methodology. J Surg Educ 74:108-116.

4. Khan R, Plahouras J, Johnston BC, Scaffidi MA, Grover SC, Walsh CM (2018) [Virtual reality simulation training for health professions trainees in gastro-intestinal endoscopy.](https://www.ncbi.nlm.nih.gov/pubmed/30117156) Cochrane Database Syst Rev 17:CD008237.

5. Walsh CM, Sherlock ME, Ling SC, Carnahan H (2012) [Virtual reality simulation training for health professions trainees in gastro-intestinal endoscopy.](https://www.ncbi.nlm.nih.gov/pubmed/22696375) Cochrane Database Syst Rev 13:CD008237.

6. Piromchai P, Avery A, Laopaiboon M, Kennedy G, O'Leary S (2015) [Virtual reality training for improving the skills needed for performing surgery of the ear, nose or throat.](https://pubmed.ncbi.nlm.nih.gov/26352008/?from_term=Piromchai+2015&from_pos=5) Cochrane Database Syst Revv 9:CD010198.

7. Harrington CM, Kavanagh DO, Quinlan JF et al (2018) Development and evaluation of a trauma decision-making simulator in Oculus virtual reality. Am J Surg 215:42-47.

8. Ziv A, Wolpe PR, Small SD, Glick S (2003) [Simulation-based medical education: an ethical imperative.](https://www.ncbi.nlm.nih.gov/pubmed/12915366) Acad Med 78:783-788.

9. Aussedat C, Robier M, Aoustin JM et al (2020) [Using virtual reality in audiological training: Our experience in 22 otolaryngology residents.](https://www.ncbi.nlm.nih.gov/pubmed/32306528) Clin Otolaryngol doi: 10.1111/coa.13554. [Epub ahead of print]

10. Kron FW, Fetters MD, Scerbo MW, et al (2017) Using a computer simulation for teaching communication skills: A blinded multisite mixed methods randomized controlled trial. Patient Educ Couns 100:748–759.

11. Soltanimehr E, Bahrampour E, Imani MM, Rahimi F, Almasi B, Moattari M (2019) [Effect of virtual versus traditional education on theoretical knowledge and reporting skills of dental students in radiographic interpretation of bony lesions of the jaw.](https://pubmed.ncbi.nlm.nih.gov/31238927/?from_term=Soltanimehr++2019&from_pos=3) BMC Med Educ 25;19:233.

12. Zhao J, Xu X, Jiang H, Ding Y (2020) [The effectiveness of virtual reality-based technology on anatomy teaching: a meta-analysis of randomized controlled studies.](https://www.ncbi.nlm.nih.gov/pubmed/32334594) BMC Med Educ 25;20:127.

13. Walsh CM, Ling SC, Wang CS, Carnahan H (2009) [Concurrent versus terminal feedback: it may be better to wait.](https://www.ncbi.nlm.nih.gov/pubmed/19907387) Acad Med 84:S54-57.

14. Salmoni AW, Schmidt RA, Walter CB (1984) Knowledge of results and motor learning: A review and critical reappraisal. Psychol Bull 95:355–386.

15. Lim, D. H. and Johnson, S. D. (2002) Trainee perceptions of factors that influence learning transfer.  International Journal of Training and Development 6:36–48

16. Alanazi AA, Nicholson N (2019) Audiology and speech-pathology simulation training on the 1-3-6 early hearing detection and intervention timeline. Am J Audiol 10;28:348-361.

17. [Alanazi AA](https://www.ncbi.nlm.nih.gov/pubmed/?term=Alanazi%20AA%5BAuthor%5D&cauthor=true&cauthor_uid=27653494), [Nicholson N](https://www.ncbi.nlm.nih.gov/pubmed/?term=Nicholson%20N%5BAuthor%5D&cauthor=true&cauthor_uid=27653494), [Atcherson SR](https://www.ncbi.nlm.nih.gov/pubmed/?term=Atcherson%20SR%5BAuthor%5D&cauthor=true&cauthor_uid=27653494), [Franklin C](https://www.ncbi.nlm.nih.gov/pubmed/?term=Franklin%20C%5BAuthor%5D&cauthor=true&cauthor_uid=27653494), [Anders M](https://www.ncbi.nlm.nih.gov/pubmed/?term=Anders%20M%5BAuthor%5D&cauthor=true&cauthor_uid=27653494), [Nagaraj N](https://www.ncbi.nlm.nih.gov/pubmed/?term=Nagaraj%20N%5BAuthor%5D&cauthor=true&cauthor_uid=27653494), [Franklin J](https://www.ncbi.nlm.nih.gov/pubmed/?term=Franklin%20J%5BAuthor%5D&cauthor=true&cauthor_uid=27653494), [Highley P](https://www.ncbi.nlm.nih.gov/pubmed/?term=Highley%20P%5BAuthor%5D&cauthor=true&cauthor_uid=27653494) (2016) Use of baby ISAO simulator and standardized parents in hearing screening and parent counseling education. [Am J Audiol](https://www.ncbi.nlm.nih.gov/pubmed/27653494) 1;25:211-223.

18. Yeh DH, Fung K, Malekzadeh S (2017) [Boot camps: Preparing for residency.](https://pubmed.ncbi.nlm.nih.gov/28669460/?from_term=Yeh+DH&from_cauthor_id=28669460&from_pos=1) Otolaryngol Clin North Am 50:1003-1013.

19. [Lambert T](https://www.ncbi.nlm.nih.gov/pubmed/?term=Lambert%20T%5BAuthor%5D&cauthor=true&cauthor_uid=32130522), [Blessberger H](https://www.ncbi.nlm.nih.gov/pubmed/?term=Blessberger%20H%5BAuthor%5D&cauthor=true&cauthor_uid=32130522), [Fellner A](https://www.ncbi.nlm.nih.gov/pubmed/?term=Fellner%20A%5BAuthor%5D&cauthor=true&cauthor_uid=32130522), [Steinwender C](https://www.ncbi.nlm.nih.gov/pubmed/?term=Steinwender%20C%5BAuthor%5D&cauthor=true&cauthor_uid=32130522), [Tekian A](https://www.ncbi.nlm.nih.gov/pubmed/?term=Tekian%20A%5BAuthor%5D&cauthor=true&cauthor_uid=32130522) (2020) Self-assessment of medical students and young physicians in training: Self-confidence of clinical competencies and working or learning environment. [Wien Klin Wochenschr](https://www.ncbi.nlm.nih.gov/pubmed/32130522) 132:155-160.

20. Maran NJ, Glavin RJ (2003) Low- to high-fidelity simulation – a continuum of medical education? Medical Education 37: 22-28.

21. Koch RW, Moodie S, Folkeard P, Scollie S, Janeteas C, Agrawal SK, Ladak HM (2019) [Face and content validity of a probe tube placement training simulator.](https://pubmed.ncbi.nlm.nih.gov/30461412/?from_term=Koch+RW&from_cauthor_id=30461412&from_pos=1) J Am Acad Audiol 30:227-234.

22. Koch RW, Saleh H, Folkeard P, Moodie S, Janeteas C, Agrawal SK, Ladak HM, Scollie S (2020) [Skill transference of a probe-tube placement training simulator.](https://www.ncbi.nlm.nih.gov/pubmed/31274072) J Am Acad Audiol 31:40-49.

23. Dzulkarnain AA, Rahmat S, Mohd Puzi NA, Badzis M (2017) [Towards developing high-fidelity simulated learning environment training modules in audiology.](https://www.ncbi.nlm.nih.gov/pubmed/28255138) Med J Malaysia 72:37-45.

24. Phelps A, Fritchle A, Hoffman H (2004) [Passive vs. active virtual reality learning: the effects on short- and long-term memory of anatomical structures.](https://www.ncbi.nlm.nih.gov/pubmed/15544293) Stud Health Technol Inform 98:298-300.

25. Ryan E, Poole C (2019) [Impact of virtual learning environment on students' satisfaction, engagement, recall, and retention.](https://www.ncbi.nlm.nih.gov/pubmed/31229502) J Med Imaging Radiat Sci 50:408-415.

**Figure legends**

Figure 1: Screenshots of the VR system for audiometric training. A: Captions for the handle functions; B: Otoscopy interpretation; C: Tone audiometry and determination of auditory thresholds; D: Speech audiometry and determination of auditory thresholds; E: Example of feedback regarding masking for speech audiometry.

Figure 2: Pre-test (open symbols) and post-test scores (filled symbols) for the TT group (blue) and the VRT group (red); data are also shown for the TT group after completing the VR training (blue squares with x). The black horizontal lines show mean scores. The asterisks indicate significant differences (* = p < 0.05; ** = 0.001<p <0.05; *** = p < 0.001).

Figure 3: Results of post-training satisfaction and self-confidence surveys for the VR and TT groups. Each bar represents the percentage of respondents for each rating. The asterisks indicate significant differences between groups (*** = p<0.001).

Figure 4: Results of VR quality surveys collected after VR training for the VR and TT groups. Each bar represents the percentage of respondents for each rating.
